# Variation in *Symbiodinium* ITS2 Sequence Assemblages among Coral Colonies

**DOI:** 10.1371/journal.pone.0015854

**Published:** 2011-01-05

**Authors:** Michael Stat, Christopher E. Bird, Xavier Pochon, Luis Chasqui, Leonard J. Chauka, Gregory T. Concepcion, Dan Logan, Misaki Takabayashi, Robert J. Toonen, Ruth D. Gates

**Affiliations:** 1 Hawai'i Institute of Marine Biology, School of Ocean and Earth Science and Technology, University of Hawai'i, Kāne'ohe, Hawai'i, United States of America; 2 Instituto de Investigaciones Marinas y Costeras “José Benito Vives de Andréis” INVEMAR. A.A., Santa Marta, Colombia; 3 Institute of Marine Sciences, University of Dar es Salaam, Zanzibar, Tanzania; 4 School of Biological Sciences, Victoria University of Wellington, Wellington, New Zealand; 5 Department of Marine Science, University of Hawai'i at Hilo, Hilo, Hawai'i, United States of America; Biodiversity Insitute of Ontario - University of Guelph, Canada

## Abstract

Endosymbiotic dinoflagellates in the genus *Symbiodinium* are fundamentally important to the biology of scleractinian corals, as well as to a variety of other marine organisms. The genus *Symbiodinium* is genetically and functionally diverse and the taxonomic nature of the union between *Symbiodinium* and corals is implicated as a key trait determining the environmental tolerance of the symbiosis. Surprisingly, the question of how *Symbiodinium* diversity partitions within a species across spatial scales of meters to kilometers has received little attention, but is important to understanding the intrinsic biological scope of a given coral population and adaptations to the local environment. Here we address this gap by describing the *Symbiodinium* ITS2 sequence assemblages recovered from colonies of the reef building coral *Montipora capitata* sampled across Kāne'ohe Bay, Hawai'i. A total of 52 corals were sampled in a nested design of Coral Colony(Site(Region)) reflecting spatial scales of meters to kilometers. A diversity of *Symbiodinium* ITS2 sequences was recovered with the majority of variance partitioning at the level of the Coral Colony. To confirm this result, the *Symbiodinium* ITS2 sequence diversity in six *M. capitata* colonies were analyzed in much greater depth with 35 to 55 clones per colony. The ITS2 sequences and quantitative composition recovered from these colonies varied significantly, indicating that each coral hosted a different assemblage of *Symbiodinium*. The diversity of *Symbiodinium* ITS2 sequence assemblages retrieved from individual colonies of *M. capitata* here highlights the problems inherent in interpreting multi-copy and intra-genomically variable molecular markers, and serves as a context for discussing the utility and biological relevance of assigning species names based on *Symbiodinium* ITS2 genotyping.

## Introduction

Coral reefs are biologically diverse ecosystems providing habitat for a wide range of marine organisms. The growth of corals and their ability to form the calcium carbonate substrate reflects their endosymbioses with photosynthetic dinoflagellates belonging to the genus *Symbiodinium* [1]. Nine divergent lineages, clades A–I, have been described in *Symbiodinium* based on nuclear ribosomal DNA (rDNA) and chloroplast 23S rDNA [2] with each clade containing multiple genetic varieties often resolved using the internal transcribed spacer (ITS) regions [e.g. [3]–[6]].

*Symbiodinium* diversity is partitioned by a variety of factors including biogeographical barriers, host species, colony depth, irradiance, and host symbiont transmission strategy [7]–[10]. Biogeographic patterns in *Symbiodinium* are evident between reefs in different oceans (Pacific versus Atlantic) [9], among reefs within an ocean (e.g. Pacific reefs in Japan and the Great Barrier Reef Australia) [11], [12], and from reefs across a latitudinal gradient (e.g. eastern Australia coastline) [12], [13]. The same coral species from inshore and offshore reefs within the same reef complex (e.g. in the central Great Barrier Reef or in Panama) can also associate with different *Symbiodinium* [12], [14], as can colonies of the same species from the same reef environment [5], [10], [14], [15]. Fidelity in the association between some coral species and *Symbiodinium* has lead to a degree of co-evolution resulting in host-symbiont specificity [9], [16]. For example, the ITS2 *Symbiodinium* genotype C42 associates with *Pocillopora* and C31 with *Montipora* [9]. Attributed to levels of irradiation, *Symbiodinium* in corals such as *Montastraea* spp. and *Madracis pharensis* in Panama [8], [17] and *Pocillopora damicornis* in the Great Barrier Reef [18] partition as a function of depth and/or location on individual colonies [8]. Host symbiont acquisition strategy also affects *Symbiodinium* assemblages with hosts that acquire their symbionts from the environment (horizontal symbiont acquisition strategy) primarily associating with a similar pool of symbionts, and those that acquire their symbionts from the parent colony (vertical symbiont acquisition strategy) harboring their own unique suite of symbionts specific to a host genus [9], [10].

Understanding the factors that affect distribution and specificity patterns in coral-dinoflagellate symbioses and the physiological range of host-symbiont combinations is important for understanding how corals will respond to environmental change. In this regard, functional variability in isolated *Symbiodinium* types and specific coral-*Symbiodinium* symbioses have been correlated with numerous factors. Variation in the photophysiology of *Symbiodinium* [17], [19], [20], growth rate of coral colonies [21], symbiont carbon fixation and translocation to the host [22], [23], symbiont thermal tolerance [24], and host disease susceptibility [22] all provide evidence for range thresholds in physiological performance of different host-symbiont assemblages as a response to the environment. As coral bleaching and disease are predicted to impact coral reef ecosystems in the future and have recently increased in severity and occurrence [25], [26], the different host-symbiont combinations that can occur and the environmental tolerance of those symbioses will provide the framework for predicting future shifts in coral reef communities.

The number of unique *Symbiodinium* that reside in individual coral hosts is an area of ongoing debate [27], [28]. Heterogeneous mixtures of *Symbiodinium* have been identified in a variety of host species e.g. [7], [8], [15], [18], and more sensitive molecular techniques such as quantitative real time PCR have enabled the detection of *Symbiodinium* clades in low abundance [29]–[31]. However, the number of *Symbiodinium* species and their occurrence among marine hosts remains a central issue that is highly relevant to our understanding of the capacity of coral-algal symbiosis and reef ecosystems to adapt with changes in the environment [32]. The nuclear internal transcribed spacer region 2 (ITS2) is currently most often utilized to resolve *Symbiodinium* diversity within the phylogenetic clades A–I e.g. [2], [5], [12], [16], [18], [33], and is being promoted as a species level marker [9], [30], [34]. However, the multi-copy nature and intra-genomic variability of the ITS2 [35], [36] often results in the isolation of more than one ITS2 sequence type from an individual *Symbiodinium* cell, and this interpretational complexity combined with low genetic divergence among ITS2 sequences [e.g. 9] makes the application of this marker in species assignment problematic [16], [37].

In order to further investigate the partitioning of *Symbiodinium* in corals and the utility of the ITS2 marker in describing *Symbiodinium* diversity, we set out to investigate the *Symbiodinium* communities in colonies of *Montipora capitata* at similar depths over a spatial scale of meters to kilometers in Kāne'ohe Bay, O'ahu Island, Hawai'i. As *M. capitata* exhibits vertical transmission of its symbionts, we also set out to examine whether patterns of *Symbiodinium* ITS2 diversity map onto the *M. capitata atpsβ* and *nad5* genotypes. The data reveal that *Symbiodinium* ITS2 diversity is different among colonies of *M. capitata* and does not reflect host genotype. These data highlight both the complexity of the *Symbiodinium* ITS2 sequence diversity in corals, and are used as a framework to discuss the problems inherent in using this marker to designate species in the genus *Symbiodinium*.

## Methods

### Ethics Statement

This study was conducted under the research guidelines of the University of Hawaii Executive Policy E5.211 and corals collected under the State of Hawaii Special Activity Permit number 2007-02 issued to the Hawaii Institute of Marine Biology.

### Sample collection and sites

**Figure 1:**
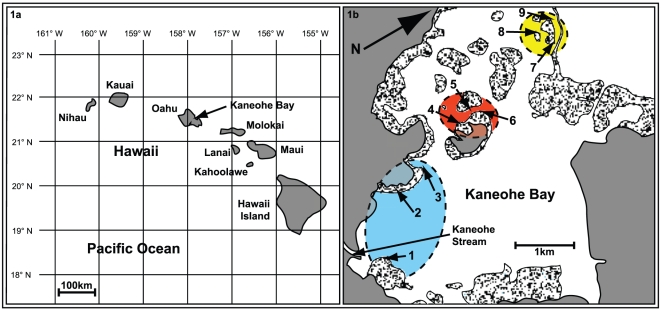
Location of corals sampled in study. Location of study in Hawai'i (1a) and sites in Kāne'ohe Bay, O'ahu (1b). Six colonies of *Montipora capitata* were sampled at a depth of 1–2 m from each of the nine sites (except Site 9 where only 4 colonies were sampled). Region 1 is shaded in blue, region 2 in green, and region 3 in yellow.

The sampling for this study was conducted in June 2007 in Kāne'ohe Bay, on the island of O'ahu. 52 colonies of *Montipora capitata* (brown branching morph) were sampled from one location at the same relative position on each colony (upper region) using a hammer and chisel at a depth of 1–2 m from three sites nested in three regions of the bay (sites 1–9; Figure 1) that lie on a northerly environmental gradient from nearshore to offshore. Region 1 was located near the Kāne'ohe Stream mouth (Sites 1–3), Region 2 in the centre of the bay (Sites 4–6), and Region 3, near the outer barrier reef (Sites 7–9). Latitudinal and longitudinal coordinates for Sites 1–9 are 21.24.902N and 157.46.826W, 21.25.271N and 157.47.255W, 21.25.574N and 157.47.336W, 21.26.039N and 157.47.497W, 21.26.200N and 157.47.518W, 21.26.265 and 157.47.440W, 21.27.026N and 157.47.585W, 21.26.992N and 157.47.762W, 21.27.112N and 157.47.820W, respectively. Six *M. capitata* colonies were sampled from Sites 1–9. Two samples from Site 9 failed to amplify in PCR, reducing the sample number at that site to four.

### DNA extraction

For extraction of nucleic acids, the coral fragments (≈5 mm^2^ of tissue from verrucae and surrounding corallites including entire polyps) were removed from each colony and stored at 4°C in 400 µl of DNA extraction buffer [50% (w/v) guanidinium isothiocyanate; 50 mM Tris pH 7.6; 10 µM EDTA; 4.2% (w/v) sarkosyl; 2.1% (v/v) β-mercaptoethanol] at the time of collection, until processed (up to 2 weeks). The coral samples in DNA extraction buffer were then incubated at 72°C for 10 min and centrifuged at 16,000 g for 5 min. The supernatant was mixed with an equal volume of 100% isopropanol to precipitate the DNA and chilled at −20°C overnight. The precipitated DNA was pelleted by centrifugation at 16,000 g for 15 min, and washed in 70% ethanol before resuspension and storage in Tris Buffer (0.1 M pH 8).

### PCR, cloning, and sequencing of *Symbiodinium*

The *Symbiodinium* partial 5.8S, ITS2, and partial 28S region was amplified in PCR using the forward *its-dino* (5′ GTGAATTGCAGAACTCCGTG 3′) and reverse *its2rev2* (5′ CCTCCGCTTACTTATATGCTT 3′) primers [38]. The products of these amplifications are referred to from here as *Symbiodinium* ITS2 sequences. Each 25 µl PCR reaction contained 1 µl of DNA template, 2.5 µl of 10x ImmoBuffer, 0.1 µl IMMOLASE™ Hot-Start DNA Polymerase (Bioline, MA), 3 mM MgCl_2_, 0.5 µl of 10 mM total dNTPs (2.5 mM each), 5 pmol each primer, and deionized sterile water to volume. PCR was performed on a BioRad iCycler™ using the following conditions: 95°C for 7 min, followed by 35 cycles of 45 s at 95°C, 45 s at 52°C, and 45 s at 72°C, with a final extension at 72°C for 5 min. PCR amplicons were purified using the QIAquick® PCR Purification Kit (Qiagen, CA), ligated into the pGEM®-T Easy vector (Promega, WI), transformed into α-select gold efficiency competent cells (Bioline, MA), and grown overnight on selective LB media (ampicillin 50 µg/ml, 0.1 mM IPTG, 50 µg/ml X-gal). Positive clones were grown overnight in Circlegrow® (MP Biomedicals, CA) and plasmids purified using the Perfectprep® Plasmid Isolation Kit (Eppendorf, Hamburg). Clones from PCR products (3 clones from 1 coral colony, 5 clones from each of 36 coral colonies, 6 from each of 13 coral colonies, and 7 from each of 2 coral colonies) were sequenced in both directions using BigDye Terminators (PerkinElmer, MA) on an ABI-3100 automated sequencer at the University of Hawai'i. Additional clones were sequenced from two colonies sampled from each region (six colonies in total, 35–55 clones per colony). Sequences were inspected, aligned, and edited using MacVector® 8.0.2 software. *Symbiodinium* ITS2 sequences used for downstream analyses were edited as described in Stat *et al.* [16]. For all analyses, *Symbiodinium* ITS2 was categorized by clade (C or D) [34], ITS2 secondary structure (folding), and ITS2 sequence. The secondary structure of all ITS2 sequences were estimated using 4SALE and the ITS2 database website [39]–[42] using published *Symbiodinium* ITS2 structures as templates [16], [36], [43].

### PCR and sequencing of *Montipora capitata* genes

To determine whether *Symbiodinium* ITS2 composition is a factor of host lineage, the host *Montipora capitata* colonies were genotyped using both the mitochondrial NADH dehydrogenase 5′ intron (*nad5*) and the nuclear ATP synthetase subunit beta intron (*atpsβ*). *M. capitata nad5* was amplified with primer pair *ND51a* (*NAD5_700F*: 5′ YTGCCGGATGCYATGGAG 3′ and *NAD1_157R*: 5′ GGGGAYCCTCATRTKCCTCG 3′) as outlined in Concepcion *et al.* [44], and *atpsβ* was amplified with a primer pair redesigned from Jarman *et al.* [45] to be specific for *M. capitata* (F: 5′ TGATTGTGTCTGGTGTAATCAGC 3′ and R: 5′ CGGGCACGGGCGCCGGGGGGTTCGTTCAT3′ ) [46]. For both markers, each 25 µl PCR contained 1 µl of DNA template, 2.5 µl of 10x ImmoBuffer, 0.1 µl IMMOLASE™ Hot-Start DNA Polymerase (Bioline Inc.), 3 mM MgCl_2_, 0.5 µl of 10 mM total dNTPs (2.5 mM each), 13 pmol each primer, and deionized sterile water to volume. PCR amplification was performed on a BioRad iCycler™ as follows: 95°C for 7 min, followed by 35 cycles at 95°C for 30 s, 53°C for 30 s, 72°C for 30 s, and a final extension at 72°C for 10 min. All successfully amplified PCR products were “cleaned” with 0.75 units of Exonuclease I: 0.5 units of Shrimp Alkaline Phosphatase (Exo:SAP) per 7.5 µl PCR product at 37°C for 60 min, followed by deactivation at 80°C for 10 min prior to being cycle-sequenced in both directions using Big Dye Terminators (Applied Biosystems) and run on an ABI-3130XL automated DNA sequencer. *atpsβ* alignments were confirmed by eye and trimmed to 252 bp. Since computational phasing of diploid nuclear loci can be more accurate than cloning in separating alleles from heterozygous individuals [47], gametic phases for *atpsβ* were inferred using Phase [48], [49] as implemented in DnaSP [50].

### Statistical parsimony networks

Statistical parsimony networks of *Symbiodinium* ITS2 sequences were constructed using the software TCS 1.21 [51]. The cladogram estimation was performed under a 95% connection limit and gaps were treated as a 5^th^ state with the alignment edited so that each indel was considered a single mutation.

### Analysis of spatial partitioning in *Symbiodinium* and *Montipora*

We set out to determine the spatial scale(s) at which *Montipora capitata* and *Symbiodinium* composition partition across Kāne'ohe Bay: meters (Coral Colony), 10's of meters (Site), and 100's to 1000's of meters (Region). Due to the sampling design, Sites are nested within Regions, denoted as Site(R), and *M. capitata* colonies are nested within Sites, denoted as Colony(S(R)). We used the PERMANOVA+1.0.2 software add-on for PRIMER 6 [52] to run three-level hierarchical analyses of molecular variance (AMOVA) [53] to test for spatial structuring. PERMANOVA+ was run using Type I sums of squares, unrestricted permutation of raw data, and significance was determined by permutation test (10,000 permutations) of the pseudo-F statistic. *Post hoc* pairwise comparisons were conducted among Regions, Sites, and Colonies using an alpha of 0.05 while controlling the family-wise false discovery rate at or below 0.05 [54]. Φ statistics (analogous to Wright's [55] F-statistics) were calculated from the PERMANOVA+ output following Excoffier *et al.* [53]. Φ ranges from 0 to 1, where 0 indicates that genetic composition among samples is identical and 1 indicates that at least one sample is completely differentiated and fixed for a single unique genetic sequence or type. We used PERMANOVA+ because the standard AMOVA software, Arlequin 3.1 [56], cannot run analyses on data sets with more than two hierarchical spatial levels with non-diploid data. PERMANOVA+ was not developed with AMOVA in mind, consequently, some calculations were required prior to and following the analysis. Prior to analysis, the AMOVA matrices of genetic distance were generated in Arlequin 3.1, the square root of each distance was taken, and the matrices were imported to PERMANOVA+. For *Symbiodinium* ITS2 and *M. capitata atpsβ* sequences, the simple pairwise genetic distance was used. For *Symbiodinium* ITS2 secondary structure, the average simple pairwise genetic distance among sequences coding for each folding group was used. For *Symbiodinium* ITS2 clades, because sequence divergence has no impact on the analysis of two categories (clade C or D), the only possible distances were zero or one.

AMOVA uses certain statistical terms and notations that carry accepted biological meanings based on loci with either two bi-parentally inherited alleles or one maternally inherited haplotype per individual. *Symbiodinium* ITS2 is a multi-copy intra-genomically variable marker and we are drawing sequences from multiple individuals of *Symbiodinium*, therefore we incorporate this assumption into our AMOVA analysis. We thereby negate any traditional biological inferences, such as the inbreeding coefficient Φ_IS_, that are calculated when each sequence represents a single haplotype or one of two alleles [55]. The lowest level of inference that can be made here for *Symbiodinium* is the variation in ITS2 sequences within Colonies(S(R)) relative to the variation among Colonies(S(R)), denoted as Φ_C(S(R))_. Φ_C(S(R))_ carries biological meaning, just not that of Φ_IS_. In the interest of clarity, we similarly avoid other standard AMOVA notation laden with biological implications such as Φ_CT_, Φ_SC_, and Φ_ST_ [53] in order to focus on the statistical inference of AMOVA in ITS2. If there is a significant difference in the ITS2 composition detected by the AMOVA, this implies that the *Symbiodinium* assemblages are partitioned, regardless of the actual number of individuals represented.

### Diversity Indices

“True diversity”, *D*, [57] was calculated using the Shannon and Weaver [58] diversity index, *H′*, as follows,
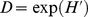
(1)

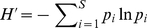
(2)where *p* is the proportion of ITS2 sequence *i* out of *s* sequences in the sample. True diversity represents the effective number of elements, which in this case is the effective number of ITS2 sequences [57]. Coverage estimates of clone libraries were calculated using the equation: 
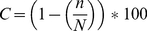
(3)where *n* is the number of unique *Symbiodinium* ITS2 sequences and *N* is the total number of clones sequenced from the library [59]. Rarefaction analyses [60], [61] were performed using Analytic Rarefaction v2 [62].

## Results

### Symbiodinium identified in Montipora capitata from Kāne'ohe Bay

**Figure 2:**
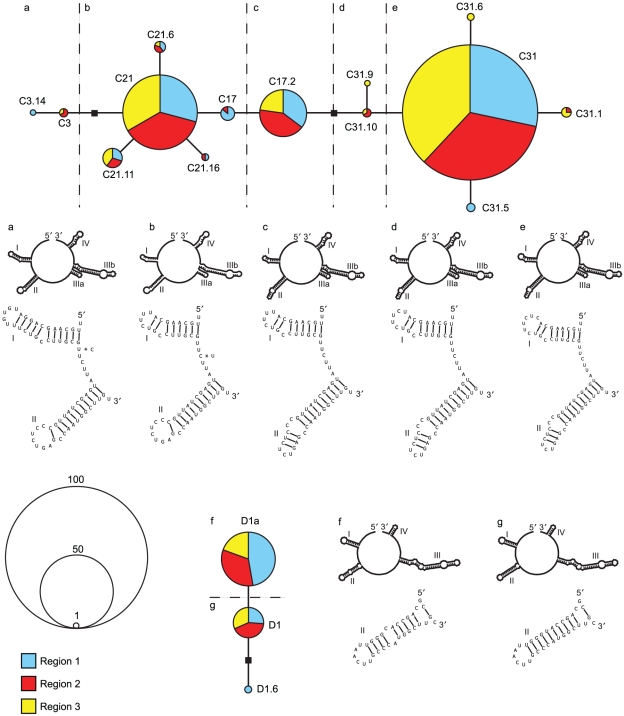
*Symbiodinium* ITS2 statistical parsimony networks for clade C and D inferred from sequences recovered from colonies of *Montipora capitata* sampled across Kāne'ohe Bay, Hawai'i. Open boxes indicate a single mutational step. Letters a – g indicate ITS2 secondary structures and dashed lines on networks separate sequences grouped by folds.

A total of 275 *Symbiodinium* ITS2 sequences belonging to clades C and D were recovered from the colonies of *M. capitata*. Seventeen different *Symbiodinium* ITS2 sequences were identified; 14 in clade C and 3 in clade D (Table 1). In addition to the previously published ITS2 sequences C3, C17, C17.2, C21, C31, D1, and D1a [5], [16], [33], [34], [63], nine novel clade C sequences and one novel clade D sequence were recovered (C3.14, C21.6, C21.11, C21.16, C31.1, C31.5, C31.6, C31.9, C31.10; and D1.6, accession numbers HQ630872-HQ630881). Statistical parsimony analysis resolved single networks for *Symbiodinium* ITS2 sequences in clade C and D (Figure 2). Conformational changes to the ITS2 secondary structures occur within stems I and II for sequences in clade C and in stem II for sequences in clade D (Figure 2, Figure S1). Five putative ITS2 folding structures were identified for sequences in clade C; Group A contains C3 and C3.14, Group B contains C17, C21, C21.6, C21.11, and C21.16, Group C contains C17.2, Group D contains C31.9 and C31.10, and Group E contains C31, C31.1, C31.5, C31.6 (Figure 2). Two folding structures were identified in clade D; Group F contains D1a, and Group G contains D1 and D1.6.

**Table 1:** *Symbiodinium* ITS2 sequences and *Montipora capitata* ATP synthetase subunit β genotypes for colonies sampled in Kāne'ohe Bay, Hawaii.

Region	Site	Colony	*Symbiodinium* ITS2 sequence(s)	*Montipora capitata* genotype
1	1	1*	D1^4^, D1a^1^	C
1		2	C21^2^, C31^2^, C21.16^1^	D
		3	D1a^3^	M
		4	C31^3^, C17.2^1^, C21.6^1^	E
		5	C31^3^, C17.2^1^, C21^1^	A
		6	C31^4^, C17.2^1^	Q
	2	7	C31^4^, C17^1^	I
		8	D1a^4^, D1^1^	A
		9*	C21^3^, C21.11^2^	I
		10	D1a^5^, D1.6^1^	R
		11	C17.2^3^, C21^2^	M
		12	D1a^4^, D1.6^1^	L
	3	13	C31^3^, C21^2^, C17^1^, C21.11^1^	H
		14	C21^3^, C17^2^, C21.6^1^	C
		15	C17.2^3^, C31.5^2^	E
		16	C31^3^, C17.2^1^, C31.5^1^	H
		17	C17^2^, C21^2^, C3.14^1^, C31^1^	A
		18	C31^4^, C17.2^1^	V
2	4	19	C17.2^2^, C31^2^, C31.10^1^	H
		20	C17.2^2^, C31^2^, C31.1^1^	J
		21	C31^2^, C3^1^, C17^1^, C17.2^1^	F
		22	C21^4^, C31^2^	P
		23	C31^3^, C17.2^2^, C21.16^1^	A
		24	C31^5^, C21^1^	H
	5	25*	D1^3^, D1a^2^	P
		26	D1a^3^, D1^2^	T
		27	C31^5^	N
		28	D1a^4^, C21^1^, D1^1^	H
		29	D1a^3^, D1^2^	G
		30	C21^2^, C21.6^2^, C17.2^1^	J
	6	31*	C21.11^3^, C21^2^	H
		32	C31^4^, C21^2^	H
		33	C21^4^, C17.2^1^	S
		34	C17.2^2^, C3^1^, C21^1^, C31.10^1^	N
		35	C31^5^, C17.2^1^	M
		36	C21^2^, C31^2^, C17.2^1^	J
3	7	37	C21^2^, C17.2^1^, C31.10^1^, D1a^1^	A
		38	C31^5^	A
		39	C31^3^, C21^2^, C31.1^1^	F
		40	C21^3^, C17.2^1^, C31^1^	B
		41	C31^3^, C21.11^1^, C31.1^1^	O
		42	C31^5^	U
	8	43	C21^2^, C17.2^1^, C21.6^1^, C31^1^, C31.6^1^	K
		44*	C31^2^, C3^1^, C21^1^, C21.11^1^	I
		45	C21^2^, C31^2^, C17.2^1^	C
		46	C17.2^2^, C31^2^, C21^1^	K
		47	D1a^4^, D1^3^	H
		48	C31^3^, C21.11^2^, C31.1^1^	W
	9	49*	C31^4^, C17.2^1^	M
		50	C31^3^, C31.6^1^, C31.9^1^	A
		51	D1^3^, D1a^2^, C31^1^	A
		52	C21^4^, C31^1^	I

*denotes corals where 35–55 *Symbiodinium* ITS2 sequences were recovered. Only the first 5 sequences identified from these colonies are presented in the table.

Superscript numerals indicate the frequency of that sequence in the colony.

### Spatial structure and diversity of *Symbiodinium* in Kāne'ohe Bay

We set out to determine if there is any partitioning of *Symbiodinium* composition at the nested scales of Region, Site(R), and host Coral Colony(S (R)) using AMOVA. In most analyses, data organized by clade, secondary structure group, or ITS2 sequence gave concordant results (Table 2), therefore we present the analyses of ITS2 sequences and note when differences occurred in secondary structure and clade analyses from here forward. Spatial partitioning of *Symbiodinium* ITS2 sequence composition was detected at the scales of Site(R) (P<0.01) and Colony(S (R)) (P<0.01; Table 2). The greatest structuring in ITS2 composition occurred among Coral Colonies(S (R)) (Φ_ C(S(R))_ = 0.87), as opposed to Sites(R) (Φ_ S(R)_ = 0.27). Because there was no spatial structure in ITS2 by Region, Regions were pooled for *post hoc* pairwise comparisons of ITS2 among all Sites and Colonies(S). Zero of 36 pairwise comparisons among Sites and 42 of 126 comparisons among Colonies(S) indicated statistically significant differences in *Symbiodinium* ITS2 sequence composition when controlling the family-wise false discovery rate, but there was no apparent spatial pattern to these differences. Among pairwise comparisons of Colonies(S), grouping the sequences by clade resulted in the detection of fewer statistically significant differences (33 of 42) than when grouping by secondary structure (42 of 42).

**Table 2:** Differences in *Symbiodinium* diversities among Colony(Site(Region)), categorized by clade, secondary structure, and ITS2 sequence, analyzed by AMOVA.

	df	Clade		ITS2 Secondary Structure		ITS2 Sequence	
		Φ	P	Φ	P	Φ	P
Region	2	−0.134	0.905	−0.126	0.925	−0.024	0.905
Site(Region)	6	0.287*	0.010*	0.268*	0.009*	0.271*	0.009*
Colony((Site)Region)	43	0.918*	0.000*	0.855*	0.000*	0.870*	0.000*

Significant values (P<0.05) are indicated with an asterisk.

**Figure 3:**
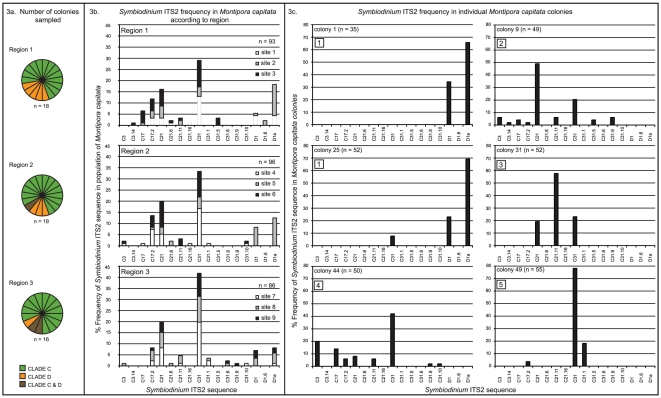
*Symbiodinium* identified in *Montipora capitata* colonies from Kāne'ohe Bay, Hawai'i. The *Symbiodinium* clades identified per region are displayed as pie charts in 3a. The frequency of *Symbiodinium* ITS2 sequences per region is displayed as bar graphs in 3b. The total frequency of ITS2 sequences per region is calculated from 3–7 clone sequences from each colony of *M. capitata* sampled in that region. The frequency of *Symbiodinium* ITS2 sequences for six colonies of *M. capitata* in which 35–55 clones were analyzed is displayed as bar graphs in 3c. Boxed numerals indicate groupings of colonies with significantly different *Symbiodinium* ITS2 composition.

**Figure 4:**
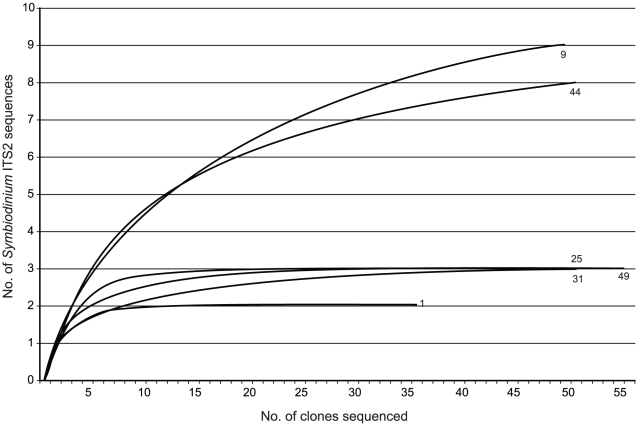
Rarefaction curves of *Symbiodinium* ITS2 sequences recovered from colonies of *Montipora capitata*. Numerals correspond to colony number from Table 1 and Figure 3.

As results from the hierarchical AMOVA indicate that the majority of the spatial structure in *Symbiodinium* ITS2 composition within *M. capitata* in Kāne'ohe Bay occurs at the scale of Coral Colony, we sequenced additional clones from two colonies haphazardly selected from each Region (6 colonies with a total of 35–55 clones per colony) to further explore inter-colony *Symbiodinium* sequence diversity. *Symbiodinium* from clade C was recovered from four colonies, clade D from one colony, and clades C and D from one colony (Figure 3). The number of sequence types recovered from each colony varied from two in Colony 1 to nine in Colony 9. The “true diversity” of *Symbiodinium* ITS2 within each colony was also variable (Colony 1: *D* = 1.9; 9: *D* = 5; 25: *D* = 2.2; 31: *D* = 2.6, 44: *D* = 5.3; 49: *D* = 1.9). AMOVA-based pairwise comparisons of ITS2 sequences in the six colonies indicate that the clone libraries from each colony are different from one another with the exception of those from Colonies 1 and 25 (Table 3). Despite the fact that all clones from Colonies 9, 31, 44, and 49 are from clade C, they represent unique non-random distributions of *Symbiodinium* ITS2 sequences. The coverage estimates indicated that the obtained sequences covered a high percentage of the diversity in each clone library (*C* = 94%, 83%, 94%, 94%, 84% and 95% for Colonies 1, 9, 25,31, 44, and 49 respectively), and are supported by rarefaction curves reaching an asymptote for libraries from four colonies (1, 25, 31, 49), and approaching an asymptote for the remaining two (9, 44; Figure 4). For Colonies 9 and 44, additional sequencing would have recovered minimally more diversity that would not have affected the result. Therefore, given that; 1) the hierarchical AMOVA indicated Coral Colony as the level at which most variation in *Symbiodinium* ITS2 sequence composition occurs, and 2) pairwise comparisons of the six colonies with increased clone sampling indicates variation in ITS2 composition between colonies, we conclude that the *Symbiodinium* assemblage in *Montipora capitata* from Kaneohe Bay is mostly partitioned at the level of Coral Colony.

**Table 3:** Φ-values for AMOVA pairwise comparisons of *Symbiodinium* ITS2 sequences among six colonies of *Montipora capitata*.

	Colony 1	Colony 9	Colony 25	Colony 31	Colony 44
Colony 9	0.982*				
Colony 25	0.041	0.904*			
Colony 31	0.988*	0.085*	0.991*		
Colony 44	0.978*	0.060*	0.901*	0.214*	
Colony 49	0.996*	0.550*	0.920*	0.704*	0.349*

Statistically significant values (α = 0.05) are indicated with an asterisk.

**Figure 5:**
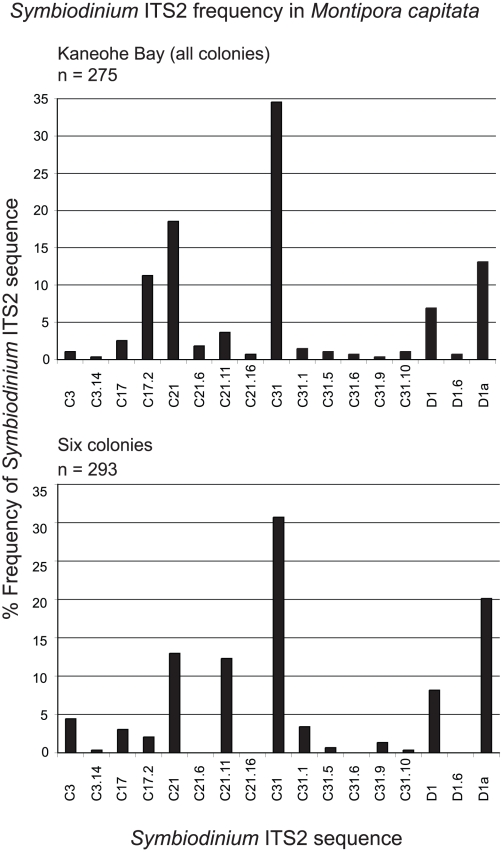
Bar graphs of *Symbiodinium* ITS2 sequences pooled from all colonies of *Montipora capitata* sampled across Kāne'ohe Bay (3–7 clones per colony), Hawai'i, and from six colonies of *M. capitata* in which 35–55 clones were analyzed.

**Figure 6:**
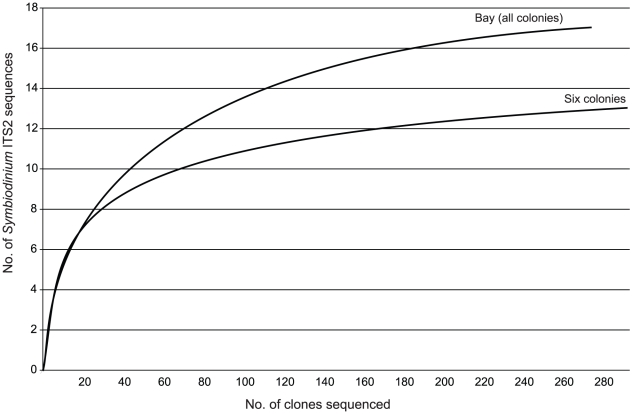
Rarefaction curves of *Symbiodinium* ITS2 sequences recovered from all colonies of *Montipora capitata* sampled in the study (3–7 clones per colony) and from six colonies of *M. capitata* in which 35–55 clones were analyzed.

The *Symbiodinium* ITS2 composition in *Montipora capitata* in Kāne'ohe Bay from all colonies (3–7 clones from 52 colonies) compared to the six colonies with additional clones (35–55 clones from 6 colonies) was assessed to determine whether a similar sequence diversity (not distribution) could be recovered using these two approaches. Of the 17 *Symbiodinium* ITS2 sequences identified in *M. capitata* from Kāne'ohe Bay, 13 were recovered from the six colonies with increased clone sequencing (Figure 5). The four that were not identified (C21.6, C21.16, C31.6, and D1.6) represent rare or low frequency in the grouped sequences. The true diversity of *Symbiodinium* ITS2 sequences was the same for all colonies sampled in the Bay grouped and the six colonies grouped (*D* = 7.2). A high coverage of sequences from the clone libraries pooled for the two groupings was achieved (*C* = 93% and 95% for all colonies and six colonies respectively) and is further supported by rarefaction analyses (Figure 6). There was also no significant difference in the *Symbiodinium* ITS2 sequence composition between the groups using AMOVA (Φ = −0.07, P = 0.487). These data suggest that the total *Symbiodinium* sequence diversity (not distribution) present in shallow water *M. capitata* in Kāne'ohe Bay can be recovered with either sequencing a few clones from many coral colonies or by sequencing a large number of clones from a few coral colonies.

### Spatial Structure of *Montipora capitata* in Kāne'ohe Bay

All corals sampled in this study had the same host *nad5* haplotype, which was identical to accession DQ351257 of *Montipora capitata* from NCBI [44]. Because there was no sequence variation among samples, this marker is not discussed any further.

Four polymorphic sites with no indels in the region aligned for *atpsβ* accounted for 11 unique alleles (Genbank accession numbers HQ630861-HQ630871) and 23 unique single-locus genotypes among our coral host samples (host genotype A–W, Table 1). We set out to determine if there is any partitioning of *Montipora capitata atpsβ* composition at the nested scales of Region, Site(R), and Colony(S (R)) using AMOVA. As we expected, there was no partitioning of *M. capitata* by Region (Φ_ CT_ = 0.01, P = 0.34) or Site(Region) (Φ_ SC_ = 0.04, P = 0.21). There was, however, a significant difference among Colonies(S(R)) (Φ_ IS_ = 0.46, P<0.001).

### Structure of Symbiodinium by Montipora capitata genotype

We tested whether *Symbiodinium* composition is related to the host coral genotype using AMOVA based on host genotypes represented in more than one colony (11 genotypes, 40 colonies). There is no indication that *Symbiodinium* ITS2 sequence composition is related to *M. capitata*'s *atpsβ* genotype (Φ = −0.14, P = 0.91).

## Discussion

### Spatial partitioning of *Symbiodinium* in *Montipora capitata* across Kāne'ohe Bay

The absence of *Symbiodinium* community structure in *Montipora capitata* among Regions in Kāne'ohe Bay contrasts with the partitioning of *Symbiodinium* in corals between oceans, reefs at different latitudes, inner and outer lagoonal environments, and on a single reef as a function of depth [e.g. [5], [7], [12], [13], [14], [17], [18]]. Differences between sites within Kāne'ohe Bay in the *Symbiodinium* community of *M. capitata* were evident primarily as a dominance of either clade C or D (colonies at Sites 2 and 5 contained more clade D than other sites). Garren *et al.* [14] reported that an increase in clade D *Symbiodinium* abundance in the *Montastraea annularis* species complex on Panamanian reefs was attributed to increased levels of suspended solids present in inner lagoonal environments relative to the outer lagoonal environment where clade C was dominant. Some symbionts in clade D *Symbiodinium* appear to be associated with corals that are exposed to “stressful” environmental conditions (e.g. elevated sea surface temperature and increased sedimentation) [64], [65]. Similarly here, Site 2 is close to the outlet of the Kāne'ohe Bay Stream and has low salinity (Palmer *et al.* unpubl. data), which may represent a stressful environment for corals at this site. However, *Symbiodinium* clade D was also more abundant than other clades at site 5, which is situated approximately 3 km from the stream outlet where there is no indication of environmental stressors (temperature, salinity, sedimentation) that are harmful to corals (Palmer *et al.* unpubl. data). Even though the presence of *Symbiodinium* clade D is mostly attributed to factors causing a more stressful environment, its occurrence may not be strictly correlated with such factors as has been shown over regional scales with temperature anomalies [66]. Also, the scale at which *Symbiodinium* diversity is recorded and the spatial scale at which environmental factors are measured may influence results investigating correlations between clade D *Symbiodinium* and stressful environments.

Spatial partitioning of *Symbiodinium* diversity in *M. capitata* across Kāne'ohe Bay was most evident at the level of Colony(S(R)). It is noteworthy that here, one coral sample was collected from a uniform location on each coral colony to allow for comparison of *Symbiodinium* assemblages among coral colonies. This strategy was adopted to minimize the sampling impact on the 52 coral colonies and to make the analytical work feasible in terms of cost and effort. However, it is possible that samples taken from multiple locations on the same coral colony might resolve spatial heterogeneity of *Symbiodinium* assemblage in *Montipora capitata* colonies, as has been demonstrated in *Montastraea* spp. from the Caribbean [7], [67]. Although very few studies examining *Symbiodinium* diversity in corals consider this issue, the complexity of *Symbiodinium* ITS2 assemblages resolved here suggest that it would be a valuable subject to examine in future studies. That said, inter-colony variation in *Symbiodinium* within the same host species has been observed over broad geographic scales (e.g. different latitudes and oceans) [9], [11], and as a function of depth on the same reef [e.g. [17], [18]]. Similarly, variation in *Symbiodinium* within the same host species within the same reef environment has been shown for a few host species [e.g. 15]. However, it has previously been reported that shallow water *M. capitata* (brown morph) around O'ahu engaged in a highly specific symbiosis with *Symbiodinium* ITS2 C31 [33]. Similarly here, ITS2 C31 was recovered from *M. capitata* colonies with the highest frequency across all Regions (Figure 3b) confirming the prevalence of *Symbiodinium* containing this ITS2 sequence. An unexpectedly high diversity of other *Symbiodinium* ITS2 sequences were also retrieved from *M. capitata* (brown morph) here, including C3, C17, C21, D1, and D1a, with some colonies containing four sub-clade C ITS2 sequences. It is important to note that these ITS2 sequences have previously been described as representing ecologically dominant endosymbionts of corals (i.e. they occupy a distinct ecological niche, either specificity to a host species or biogeographic region and hence interpreted as different species) based on fingerprint profiles of amplified *Symbiodinium* ITS2 using denaturing gradient gel electrophoresis (DGGE) from colonies sampled in nature [5], [9], [34], [63]. This high number of potential endosymbiont “species” within individual coral colonies previously reported to contain a single specific endosymbiont “species” highlights the fact that additional sampling, and/or the application of different analytical methods significantly influences the interpretation of the taxonomic nature and composition of *Symbiodinium* diversity in individual coral colonies and species. In this context, a greater understanding of the spatial scale at which *Symbiodinium* ITS2 sequences vary (among and within colonies, and among polyps from the same colony), and the extent of intra-genomic variation in individual *Symbiodinium* cells is needed.

The forces driving differences in *Symbiodinium* assemblages among the *M. capitata* colonies described here are unknown, but likely reflect some combination of host-symbiont specificity, environmental, and stochastic processes [68]. Although no evidence of specificity between *Symbiodinium* ITS2 and host mitochondrial NADH dehydrogenase 5′ intron (*nad5*) and nuclear ATP synthetase subunit beta intron (*atpsβ*) genotypes was detected, it is possible that alternate host (or *Symbiodinium*) markers with different taxonomic resolution might reveal a correlation between host genotype and their endosymbiont communities.

### Interpreting *Symbiodinium* diversity using ITS2

Identifying heterogeneous *Symbiodinium* communities is relatively easy at the cladal level because the high level of genetic variation that exists between lineages allows their presence (in high or low abundance) to be determined using sensitive molecular techniques such as Quantitative Real Time PCR [e.g. [29]–[31]]. However, defining the number of sub-clade *Symbiodinium* present in heterogeneous endosymbiotic communities using a marker like ITS2 is not as straightforward. ITS2 is a multi-copy marker that is intra-genomically variable within *Symbiodinium* [35], [36]. In an attempt to overcome these issues, the dominance of an ITS2 sequence amplified in PCR and the accompanying DGGE fingerprint is currently being used to describe the *Symbiodinium* type present in a sample and delineate species within the genus [e.g. [5], [9], [12], [30], [33], [63], [69]]. This methodology and interpretation emphasizes dominance of a sequence in a sample and disregards low abundant sequences (<5–10% in abundance) as intra-genomic variants that are not important [18], [29], [70]. However, in addition to the dominant sequence type C31, many of the *M. capitata* colonies in this study associated with multiple *Symbiodinium* ITS2 sequences that have previously been described as ecologically dominant and representative of independent biological entities (i.e. species). The most extreme examples of this are *M. capitata* colonies 9 and 44 (Table 1, Figure 3) harboring *Symbiodinium* ITS2 C3, C17, C21, C31, and other novel types, that collectively encompass almost all of the secondary structures in ITS2 recovered here. As the statistical parsimony network of clade C *Symbiodinium* depicts a step-wise evolution from the ancestral clade C sequence, ITS2 C3 [9], to the most derived, C31, and as the rDNA is multicopy and is variable in a *Symbiodinium* genome [35], [36], there are three possible biological interpretations of the sequence diversity recovered here that lie at the extremes and at some point along the continuum from intra-genomic to inter-genomic diversity. The first is that every sequence recovered represents an individual *Symbiodinium* cell type or species (i.e. the highest *Symbiodinium* diversity possible). The second is that the corals contain a single *Symbiodinium* cell type or one species that contains intra-genomic variants encompassing all the sequence diversity recovered (C3 to C31; i.e. the lowest *Symbiodinium* diversity possible). The third, and in our opinion the most likely, is some combination of possibilities 1 and 2. With the data in hand, it is impossible to distinguish which of these scenarios explains the *Symbiodinium* sequence diversity in *M. capitata* reported here. We can say, however, that because the *Symbiodinium* ITS2 sequence composition among colonies is variable, the *Symbiodinium* communities in these corals are different. The problems of interpreting exactly what the endosymbiotic ITS2 sequence data from an individual coral means in terms of species diversity are well illustrated when considering the recently nominated species *Symbiodinium trenchi* and *Symbiodinium glynni* [30], [69]. The species *Symbiodinium trenchi* is identified using the ITS2 D1a DGGE fingerprint, however, this fingerprint always contains a band that corresponds to the D1 sequence. The D1 sequence can occur independently of D1a, and when D1a is absent, the D1 DGGE fingerprint is used to define the species *Symbiodinium glynni*. A study by Thornhill *et al.* [36], however, clearly demonstrates that the D1 and D1a sequences are intra-genomic variants in an isoclonal cell line. Therefore, when the D1a ITS2 DGGE fingerprint (with its companion D1 sequence) is detected in an endosymbiotic sample, it is impossible to distinguish whether these sequences represent intra-genomic variants of one cell type, or co-occurring populations of two *Symbiodinium* species, *S. trenchi* and *S. glynni*. Thus, the use of ITS2 sequences that are known to be intra-genomic variants to delineate different species is problematic when assessing the diversity of species in endosymbiotic *Symbiodinium* communities in corals.

That said, defining cryptic *Symbiodinium* types and their prevalence is fundamentally important when considering endosymbiont shifting/shuffling in corals as a response to changes in the environment [32], [64], [71]. One solution to the problems encountered in interpreting ITS2 diversity in environmental samples (ie. host organisms) of *Symbiodinium* is to develop and apply a new marker(s) that has a similar level of resolution to the ITS2, but that exhibits a one to one relationship between sequence type and an individual *Symbiodinium* cell. In our opinion, the power of applying DGGE of *Symbiodinium* ITS2 to coral endosymbionts lies in comparing fingerprint patterns among samples to determine whether or not the signatures are the same or different, an approach widely used in the field of microbial ecology. However, the properties of ITS2 as a marker clearly make it a suboptimal choice for species assignment in *Symbiodinium*.

Endemicity and distribution ranges of *Symbiodinium* types have mostly been inferred using the ITS2 in studies generally constituting 1–2 colonies per host species [e.g. [5], [9], [12], [63]]. The utility of small host sample sizes is to enable a ”snapshot” of *Symbiodinium* diversity from various host species from numerous reef environments. However, replicate sampling of host species on reefs previously targeted in “snapshot” *Symbiodinium* diversity studies often reveal missed diversity among endosymbiont communities within a host. For example, *Pocillopora damicornis*, *Stylophora pistillata*, *Acropora palifera* and *Goniastrea favulus* have all been shown to associate with a higher diversity of *Symbiodinium* than originally perceived around Heron Island in the Great Barrier Reef [5], [10], [18], as was *Porites lobata* in Hawai'i [72], and *Montastraea franksi* and *Siderastrea siderea* in the Caribbean [73]. Similarly, a *Symbiodinium* ITS2 sequence previously considered to be Caribbean-specific was reported from *Acropora* at Johnston Atoll in the central Pacific [16]. *Symbiodinium* ITS2 C17 and C21 were not previously reported from marine invertebrates hosts in Hawai'i [33], yet they were all recovered here from increased sampling of one host species, at a single depth, from a single bay. As such, some of the generalized biogeographic and host specificity patterns of *Symbiodinium* may simply reflect a gross under-sampling of endosymbiont communities in marine invertebrates [9]. The higher *Symbiodinium* diversity and among colony endosymbiont variation shown here and in the studies described above, shows that some of the biogeographic patterns in *Symbiodinium* distribution and host specificity do not hold with increased sampling effort. As such, a much greater depth of sampling on a global scale will be required to accurately describe radiation within the genus, understand host specificity and the environmental thresholds of symbioses, and define biogeographic patterns in *Symbiodinium* diversity.

### Sampling strategy to recover *Symbiodinium* diversity

The high sequence diversity of *Symbiodinium* reported here from colonies of *Montipora capitata* was recovered by screening a small number of clones from a large number of colonies, or the inverse, screening a large number of clones from a small number of colonies. When additional parameters are included in the experimental design (e.g. sampling, depth, multiple hosts, larger biogeographic region), a greater number of colonies will need to be investigated. Also, we show that there is no standard number of *Symbiodinium* ITS2 clones that need to be sequenced from all clone libraries to accurately assess endosymbiont diversity in *M. capitata* colonies. For some colonies (e.g. Colony 1, 25, 31, 49; Figure 4) *Symbiodinium* ITS2 diversity can be captured with <10 clone sequences, while for others (e.g. Colony 9 and 44) a higher number of clones need to be sequenced to get an accurate estimation of endosymbiont ITS2 diversity. Similarly, Stat *et al.* [16] showed that only *Symbiodinium* ITS2 C15 was recovered from *Porites lobata* at Johnston atoll, while a higher sequence diversity (2–7 sequences) was recovered in other coral species at the same location. Therefore the number of coral colonies analyzed and number of clones sequenced per colony will need to be tailored to each study and will reflect some combination of the host species investigated and the environment from which the coral was sampled.

### Conclusion

*Symbiodinium* ITS2 sequence assemblages found in *M. capitata* are variable among individual colonies. The driving force behind these differences is unknown, but likely reflects some combination of host-symbiont specificity, environmental, and stochastic processes. The multi-copy nature and known variability of ITS2 within individual *Symbiodinium* cells (intra-genomic) make it impossible to distinguish how many independent biological entities these sequence assemblages represent. However, the intricacy of this dataset highlights both the complexity of coral *Symbiodinium* associations, and innate problems in interpreting ITS2 sequence types that question the assumptions and validity of using the ITS2 to delineate *Symbiodinium* species.

## Supporting Information

Figure S1 Symbiodinium ITS2 secondary structures.
(DOC)

## References

[1] Muscatine L, McCloskey LR, Marian RE (1981). Estimating the daily contribution of carbon from zooxanthellae to
coral animal respiration.. Limnol Oceanogr.

[2] Pochon X, Gates RD (2010). A new *Symbiodinium* clade (Dinophyceae) from
soritid foraminifera in Hawai'i.. Mol Phylogenet Evol.

[3] van Oppen MJH, Palstra FP, Piquet MT, Miller DJ (2001). Patterns of coral-dinoflagellate associations in
*Acropora*: significance of local availability and
physiology of *Symbiodinium* strains and host-symbiont
selectivity.. P Roy Soc B-Biol Sci.

[4] van Oppen MJH, Mahiny AJ, Done TJ (2005). Geographic distribution of zooxanthella types in three coral
species on the Great Barrier Reef sampled after the 2002 bleaching
event.. Coral Reefs.

[5] LaJeunesse TC, Loh WKW, van Woesik R, Hoegh-Guldberg O, Schmidt GW (2003). Low symbiont diversity in southern Great Barrier Reef corals,
relative to those of the Caribbean.. Limnol Oceanogr.

[6] Pochon X, Garcia-Cuetos L, Baker AC, Castella E, Pawlowski J (2007). One-year survey of a single Micronesian reef reveals
extraordinarily rich diversity of *Symbiodinium* types in
soritid foraminifera.. Coral Reefs.

[7] Rowan R, Knowlton N (1995). Intraspecific diversity and ecological zonation in coral-algal
symbiosis.. P Natl Acad Sci USA.

[8] Rowan R, Knowlton N, Baker A, Jara J (1997). Landscape ecology of algal symbionts creates variation in
episodes of coral bleaching.. Nature.

[9] LaJuenesse TC (2005). “Species” radiations of symbiotic dinoflagellates in
the Atlantic and Indo-Pacific since the Miocene-Pliocene
transition.. Mol Biol Evol.

[10] Stat M, Loh WKW, Hoegh-Guldberg O, Carter DA (2008). Symbiont acquisition strategy drives host-symbiont associations
in the southern Great Barrier Reef.. Coral Reefs.

[11] Loh WKW, Loi T, Carter D, Hoegh-Guldberg O (2001). Genetic variability of the symbiotic dinoflagellates from the
wide ranging coral species *Seriatopora hystrix* and
*Acropora longicyathus* in the Indo-West
Pacific.. Mar Ecol Prog Ser.

[12] LaJeunesse TC, Bhagooli R, Hidaka M, deVantier L, Done T (2004). Closely related *Symbiodinium* spp. differ in
relative dominance in coral reef host communities across environmental,
latitudinal and biogeographic gradients.. Mar Ecol Prog Ser.

[13] Rodriguez-Lanetty M, Loh W, Carter D, Hoegh-Guldberg O (2001). Latitudinal variability in symbiont specificity within the
widespread scleractinian coral *Plesiastrea
versipora*.. Mar Biol.

[14] Garren M, Walsh SM, Caccone A, Knowlton N (2006). Patterns of association between *Symbiodinium* and
members of the *Montastraea annularis* species complex on
spatial scales ranging from within colonies to between geographic
regions.. Coral Reefs.

[15] Jones A, Berkelmans R, van Oppen MJH, Mieog JC, Sinclair W (2008). A community change in the algal endosymbionts of a scleractinian
coral following a natural bleaching event: field evidence of
acclimitization.. P Roy Soc B-Biol Sci.

[16] Stat M, Pochon X, Cowie ROM, Gates RD (2009). Specificity in communities of *Symbiodinium* in
corals from Johnston Atoll.. Mar Ecol Prog Ser.

[17] Frade PR, Englebert N, Faria J, Visser PM, Bak RPM (2008). Distribution and photobiology of *Symbiodinium*
types in different light environments for three colour morphs of the coral
*Madracis pharensis*: is there more to it than total
irradiance?. Coral Reefs.

[18] Sampayo EM, Franceschinis L, Hoegh-Guldberg O, Dove S (2007). Niche partitioning of closely related symbiotic
dinoflagellates.. Mol Ecol.

[19] Iglesias-Prieto R, Beltran VH, LaJeunesse TC, Reyes-Bonilla H, Thome PE (2004). Different algal symbionts explain the vertical distribution of
dominant reef corals in the Eastern Pacific.. P Roy Soc B-Biol Sci.

[20] Abrego D, Ulstrup KE, Willis BL, van Oppen MJH (2008). Species-specific interactions between algal symbionts and coral
hosts define their bleaching response to heat and light
stress.. P Roy Soc B-Biol Sci.

[21] Little AF, van Oppen NJH, Willis BL (2004). Flexibility in algal endosymbioses shapes growth in reef
corals.. Science.

[22] Stat M, Morris E, Gates RD (2008). Functional diversity in coral-dinoflagellate
symbiosis.. P Natl Acad Sci USA.

[23] Cantin NE, van Oppen MJH, Willis BL, Mieog JC, Negri AP (2009). Juvenille corals can acquire more carbon from high-performance
algal symbionts.. Coral Reefs.

[24] Rowan R (2004). Thermal adaptations in reef coral symbionts.. Nature.

[25] Hoegh-Guldberg O (1999). Climate change, coral bleaching and the future of the
world's coral reefs.. Marine Freshwater Research.

[26] Hughes TP, Baird AH, Bellwood DR, Card M, Connolly SR (2003). Climate change, human impacts, and the resilience of coral
reefs.. Science.

[27] Goulet TL (2006). Most corals may not change their symbionts.. Mar Ecol Prog Ser.

[28] Baker AC, Romanski AM (2007). Multiple symbiotic partnerships are common in scleractinian
corals, but not in octocorals: comment on Goulet (2006).. Mar Ecol Prog Ser.

[29] Mieog JC, van Oppen JH, Cantin NE, Stam WT, Olsen JL (2007). Real-time PCR reveals high incidence of
*Symbiodinium* clade D at low levels in four
scleractinian corals across the Great Barrier Reef: implications for
symbiont shuffling.. Coral Reefs.

[30] LaJeunesse TC, Smith RT, Finney J, Oxenford H (2009). Outbreak and persistence of opportunistic dinoflagellates during
the 2005 Caribbean mass coral “bleaching” event.. P Roy Soc B-Biol Sci.

[31] Correa A, McDonald D, Baker AC (2009). Development of clade-specific *Symbiodinium*
primers for quantitative PCR (qPCR) and their application to detecting clade
D symbionts in Caribbean corals.. Mar Biol.

[32] Buddemeier RW, Fautin DG (1993). Coral bleaching as an adaptive mechanism.. Bioscience.

[33] LaJeunesse TC, Thornhill DJ, Cox EF, Stanton FG, Fitt WK (2004). High diversity and host specificity observed among symbiotic
dinoflagellates in reef communities from Hawaii.. Coral Reefs.

[34] LaJeunesse TC (2001). Investigating the biodiversity, ecology, and phylogeny of
endosymbiotic dinoflagellates in the genus *Symbiodinium*
using the ITS region: in search of a “species” level
marker.. J Phycol.

[35] Long EO, Dawid IB (1980). Repeated genes in eukaryotes.. Annu Rev Biochem.

[36] Thornhill DJ, LaJeunesse TC, Santos SR (2007). Measuring rDNA diversity in eukaryotic microbial systems: how
intragenomic variation, pseudogenes, and PCR artifact confound biodiversity
estimates.. Mol Ecol.

[37] Correa AMS, Baker AC (2009). Understanding diversity in coral-algal symbiosis: a cluster-based
approach to interpreting fine-scale genetic variation in the genus
*Symbiodinium*.. Coral Reefs.

[38] Pochon X, Pawlowski J, Zaninetti L, Rowan R (2001). High genetic diversity and relative specificity among
*Symbiodinium*-like endosymbiotic dinoflagellates in
soritid foraminiferans.. Mar Biol.

[39] Schultz J, Müller T, Achtziger M, Seibel PN, Dandekar T (2006). The internal transcribed spacer 2 database – a web server
for (not only) low level phylogenetic analyses.. Nucleic Acids Res.

[40] Seibel PN, Müller T, Dandekar T, Schultz J, Wolf M (2006). 4SALE: a tool for synchronous RNA sequence and secondary
structure alignments and editing.. BMC Bioinformatics.

[41] Seibel PN, Müller T, Dandekar T, Wolf M (2008). Synchronous visual analysis and editing of RNA sequence and
secondary structure alignments in 4SALE.. BMC Res Notes.

[42] Selig C, Wolf M, Müller T, Dandekar T, Schultz J (2008). The ITS2 database II: homology modeling RNA structure for
molecular systematics.. Nucelic Acids Res.

[43] Hunter RL, LaJeunesse TC, Santos SR (2007). Structure and evolution of the rDNA internal transcribed spacer
(ITS) region 2 in the symbiotic dinoflagellates
(*Symbiodinium*, Dinophyta).. J Phycol.

[44] Concepcion G, Medina M, Toonen RJ (2006). Non-coding mitochondrial loci for corals.. Mol Ecol Notes.

[45] Jarman SN, Ward RD, Elliot NG (2002). Oligonucleotide primers for PCR amplification of coelomate
introns.. Mar Biotechnol.

[46] Concepcion GT, Crepeau MW, Wagner D, Kahng SE, Toonen RJ (2008). An alternative to ITS, a hypervariable, single-copy nuclear
intron in corals, and its use in detecting cryptic species within the
octocoral genus *Carijoa*.. Coral Reefs.

[47] Harrigan RJ, Mazza ME, Sorenson MD (2008). Computation vs. cloning: evaluation of two methods for haplotype
determination.. Mol Ecol Res.

[48] Stephens M, Smith N, Donnelly P (2001). A new statistical method for haplotype reconstruction from
population data.. Am J Hum Genet.

[49] Stephens M, Donnelly P (2003). A comparison of Bayesian methods for haplotype reconstruction
from population genetic data.. Am J Hum Genet.

[50] Librado P, Rozas J (2009). DnaSP v5: A software for comprehensive analysis of DNA
polymorphism data.. Bioinformatics.

[51] Clement M, Posada D, Crandall KA (2000). TCS: a computer program to estimate gene
genealogies.. Mol Ecol.

[52] Clarke KR, Warwick RM (2001). Change in marine communities: an approach to statistical analysis
and integration, 2^nd^ edn.. PRIMER-E, Plymouth.

[53] Excoffier L, Smouse PE, Quattro JM (1992). Analysis of molecular variance inferred from metric distances
among DNA haplotypes: application to human mitochondrial DNA restriction
data.. Genetics.

[54] Benjamini Y, Krieger AM, Yekutieli D (2008). Adaptive linear step-up procedures that control the false
discovery rate.. Biometrika.

[55] Wright S (1943). Isolation by distance.. Genetics.

[56] Excoffier L, Laval G, Schneider S (2005). Arlequin ver. 3.0: An integrated software package for population
genetics data analysis.. Evol Bioinform Online.

[57] Jost L (2007). Partitioning diversity into independent alpha and beta
components.. Ecology.

[58] Shannon CE, Weaver W (1963). The mathematical theory of communication..

[59] Good IJ (1953). The population frequencies of Species and the estimation of
population parameters.. Biometrika.

[60] Hurlbert SH (1971). The non-concept species diversity: a critique and alternative
parameters.. Ecology.

[61] Heck KL, van Belle G, Simberloff D (1975). Explicit calculation of the rarefaction diversity measurements
and the determination of sufficient sample size.. Ecology.

[62] Holland SM (2009). http://www.huntmountainsoftware.com.

[63] LaJeunesse TC (2002). Diversity and community structure of symbiotic dinoflagellates
from Caribbean coral reefs.. Mar Biol.

[64] Toller WW, Rowan R, Knowlton N (2001). Zooxanthellae of the *Montastraea annularis*
species complex: patterns of distribution of four taxa of
*Symbiodinium* on different reefs and across
depths.. Biol Bull.

[65] Baker AC Starger CJ, McClanahan TR, Glynn PW (2004). Corals' adaptive response to climate change.. Nature.

[66] Oliver TA, Palumbi SR (2009). Distributions of stress-resistant coral symbionts match
environmental patterns at local but not regional scales.. Mar Ecol Prog Ser.

[67] Kemp DW, Fitt WK, Schmidt GW (2008). A microsampling method for genotyping coral
symbionts.. Coral Reefs.

[68] Correa AMS, Baker AC (2010). ‘Disaster taxa’ in microbially-mediated metazoans:
how endosymbionts and environmental catastrophes influence the adaptive
capacity of reef corals.. Global Change Biol.

[69] Lajeunesse TC, Smith R, Walther M, Pinzón J, Pettay DT (2010). Host-symbiont recombination versus natural selection in the
response of coral-dinoflagellate symbioses to environmental
disturbance.. P Roy Soc B-Biol Sci.

[70] LaJeunesse TC, Pinzon JH (2007). Screening intragenomic rDNA for dominant variants can provide a
consistent retrieval of evolutionary persistent ITS (rDNA)
sequences.. Mol Phylogenet Evol.

[71] Stat M, Carter D, Hoegh-Guldberg O (2006). The evolutionary history of *Symbiodinium* and
scleractinian hosts - Symbiosis, diversity, and the effect of climate
change.. Perspect Plant Ecol.

[72] Apprill AM, Gates RD (2007). Recognizing diversity in coral symbiotic dinoflagellate
communities.. Mol Ecol.

[73] Correa AMS, Brandt ME, Smith TB, Thornhill DJ, Baker AC (2009). *Symbiodinium* associations with diseased and
healthy corals.. Coral Reefs:.

