# Supplementary material for: Variation in Symbiodinium ITS2 Sequence Assemblages among Coral Colonies
Source: PLoS One. 2011 Jan 5;6(1):e15854. doi: 10.1371/journal.pone.0015854 (PMC3016399; doi:10.1371/journal.pone.0015854)

*Symbiodinium* ITS2 secondary structures

C3 Δ*G* -52.10 kcal/mol


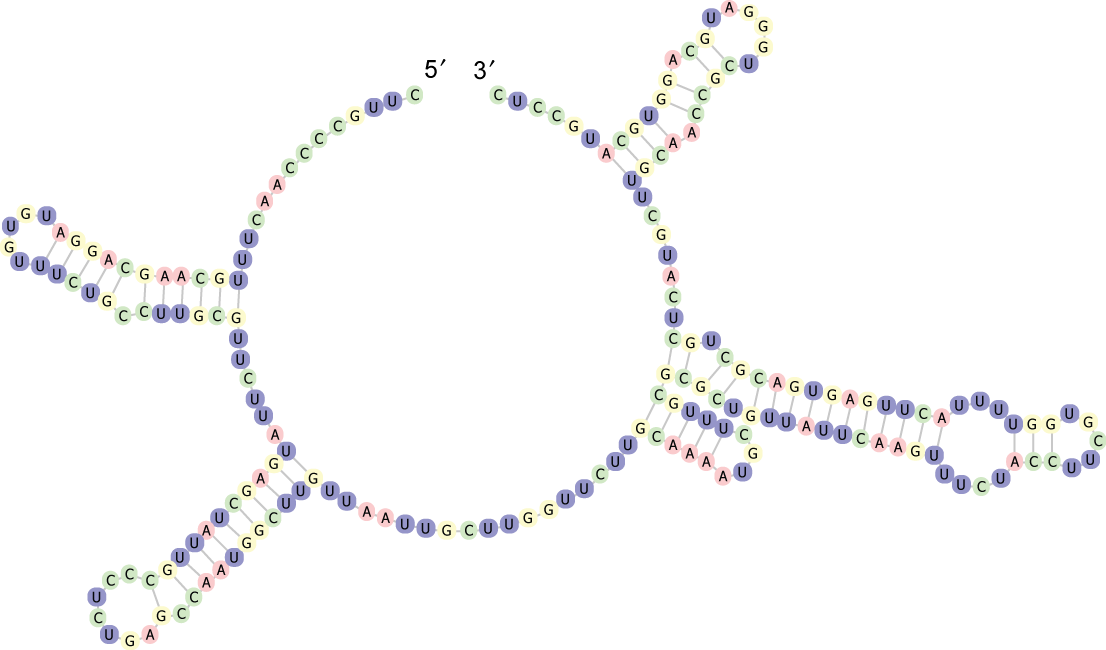


C3.14 Δ*G* -52.00 kcal/mol


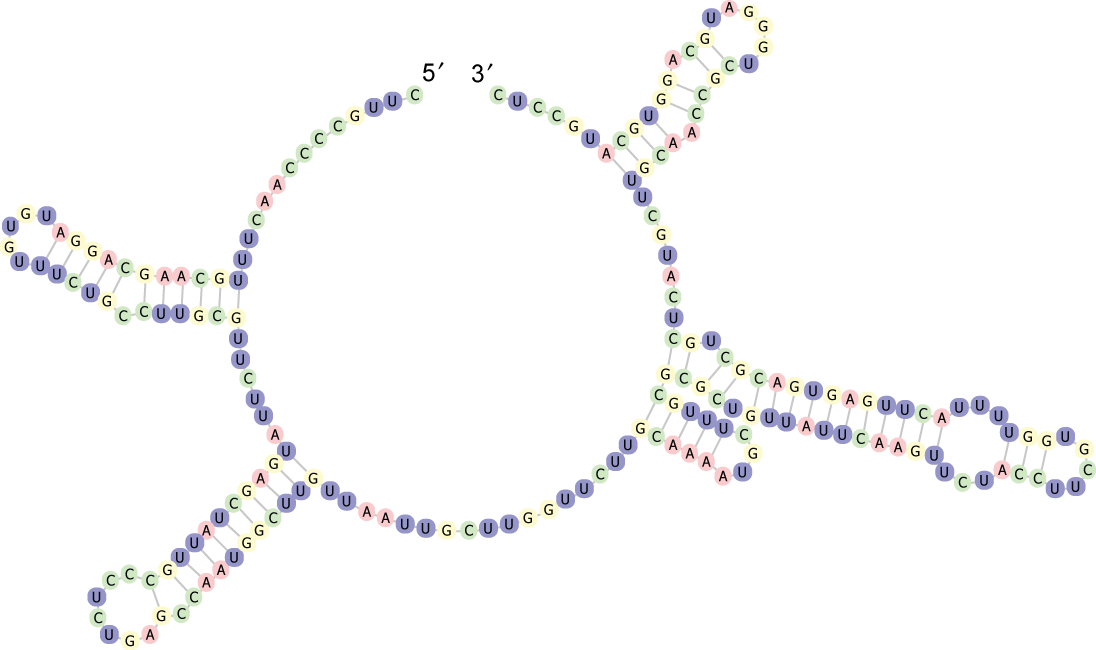


C17 Δ*G* -46.40 kcal/mol


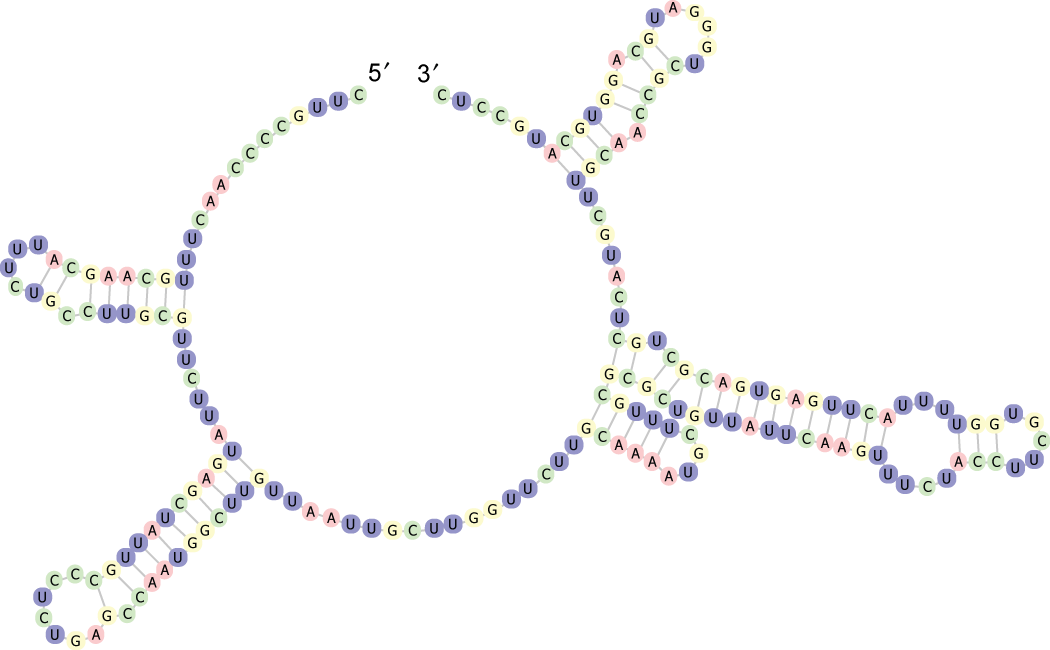


C17.2 Δ*G* -48.00 kcal/mol


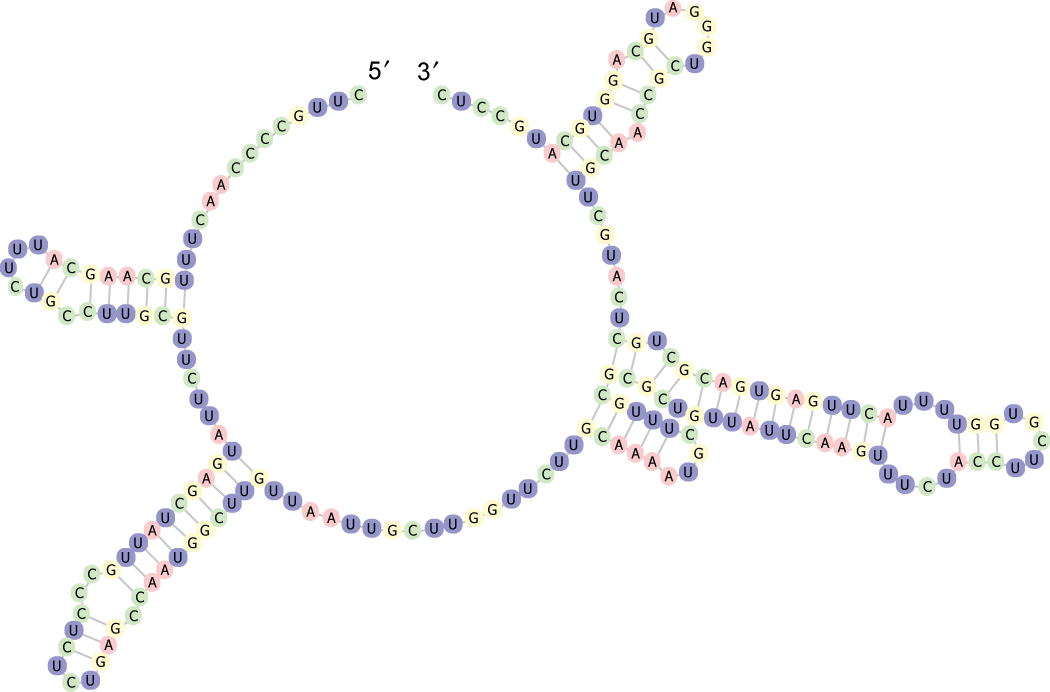


C21 Δ*G* -46.40 kcal/mol


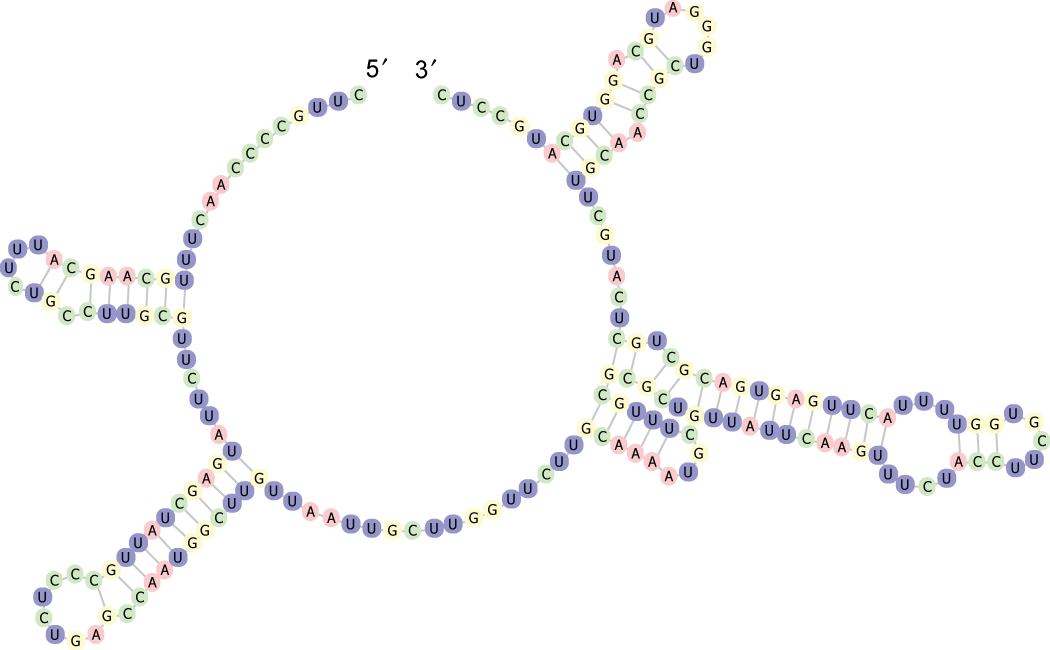


C21.6 Δ*G* -46.40 kcal/mol


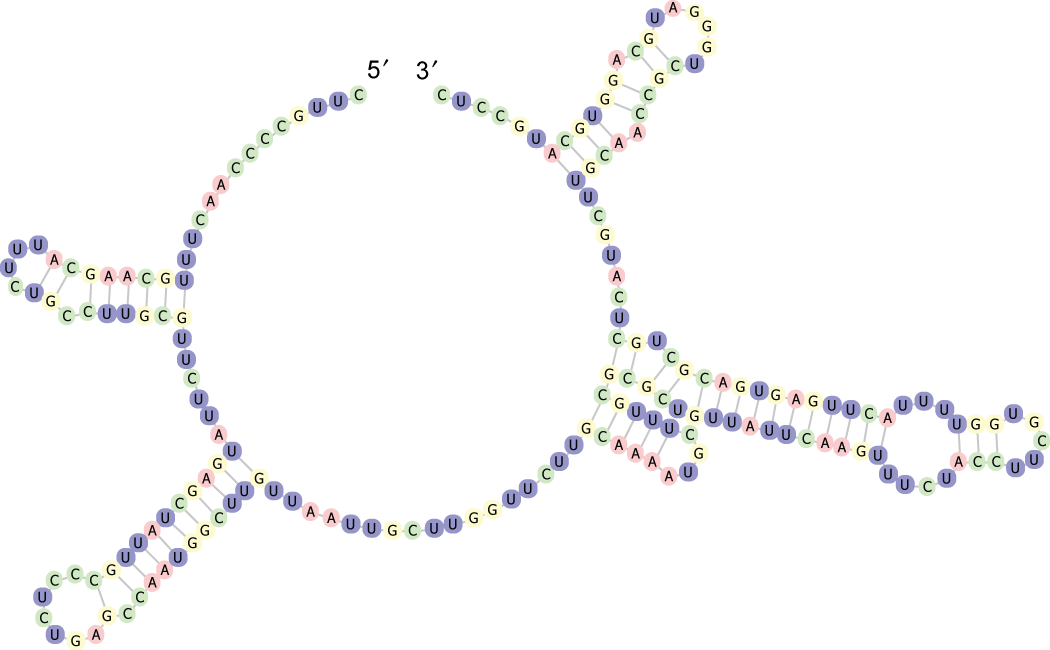


C21.11 Δ*G* -46.40 kcal/mol


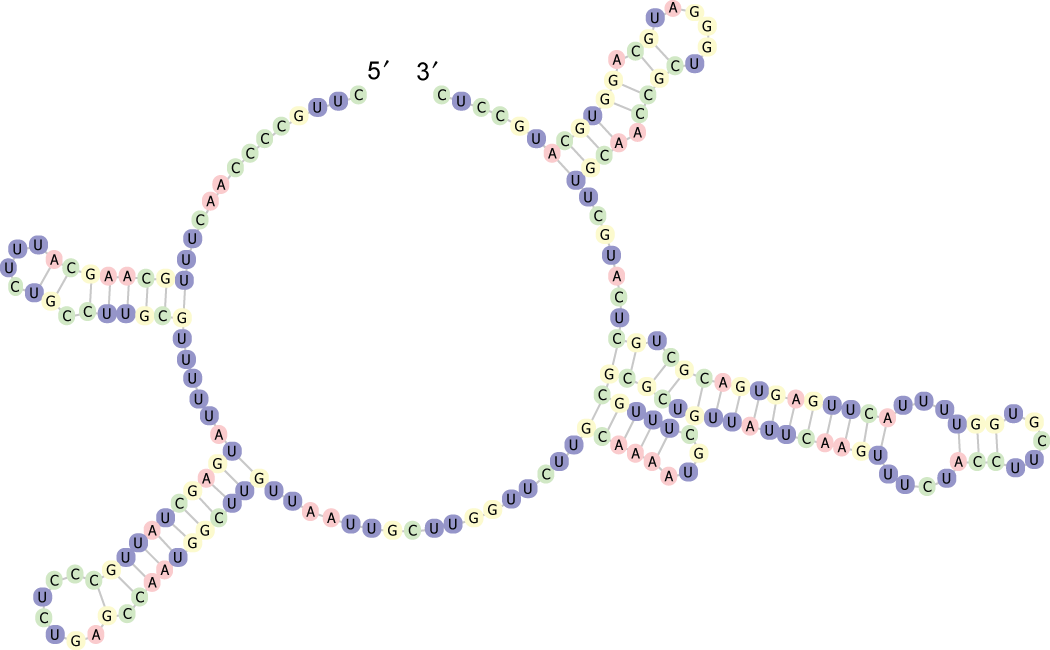


C21.16 Δ*G* -44.50 kcal/mol


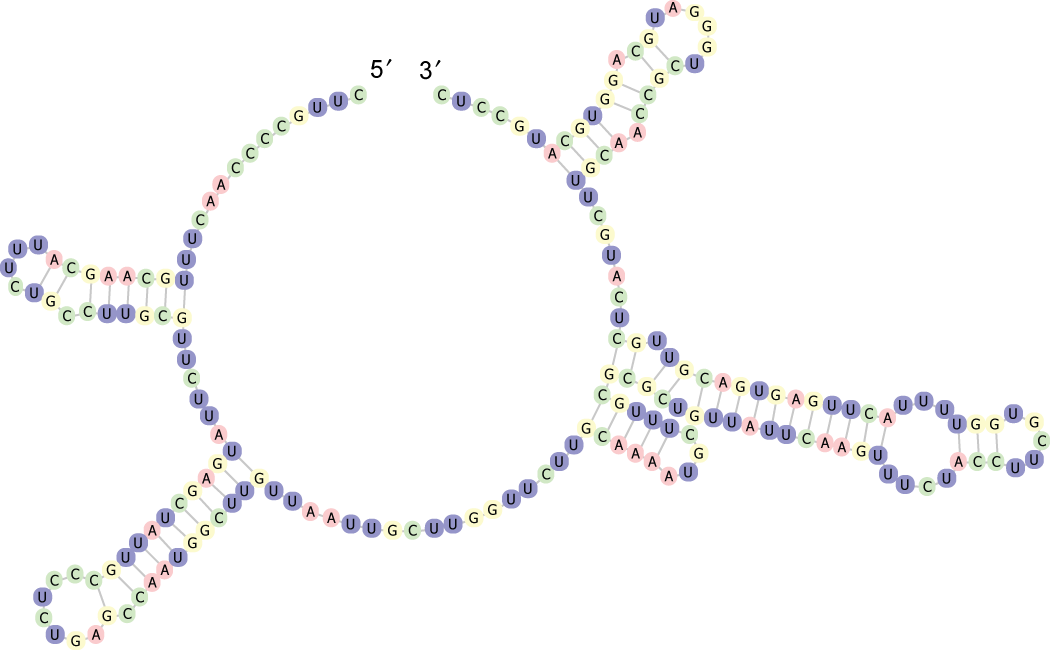


C31 Δ*G* -49.30 kcal/mol (Hunter et al 2007)


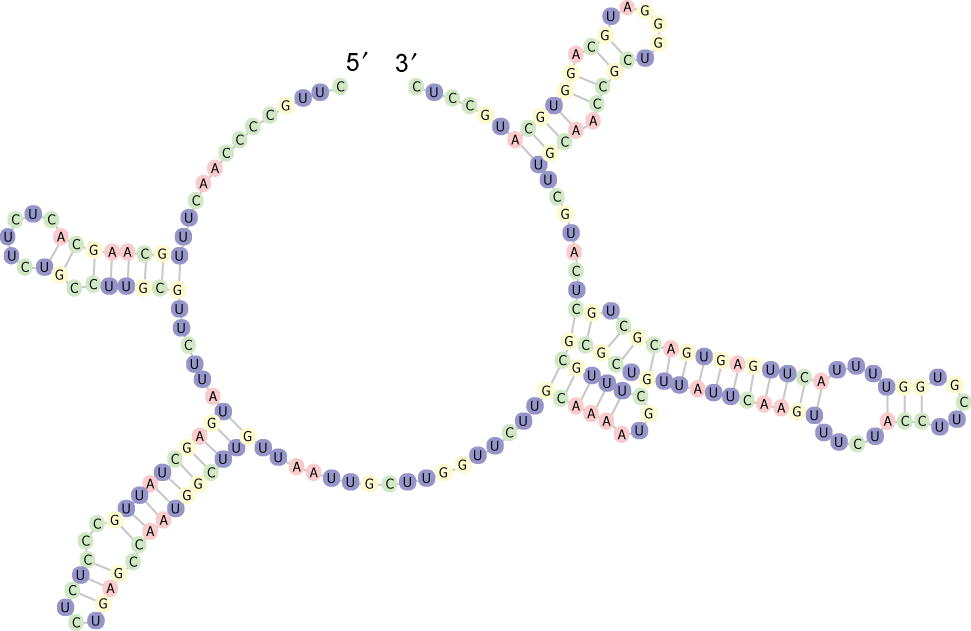


C31.1 Δ*G* -48.10 kcal/mol


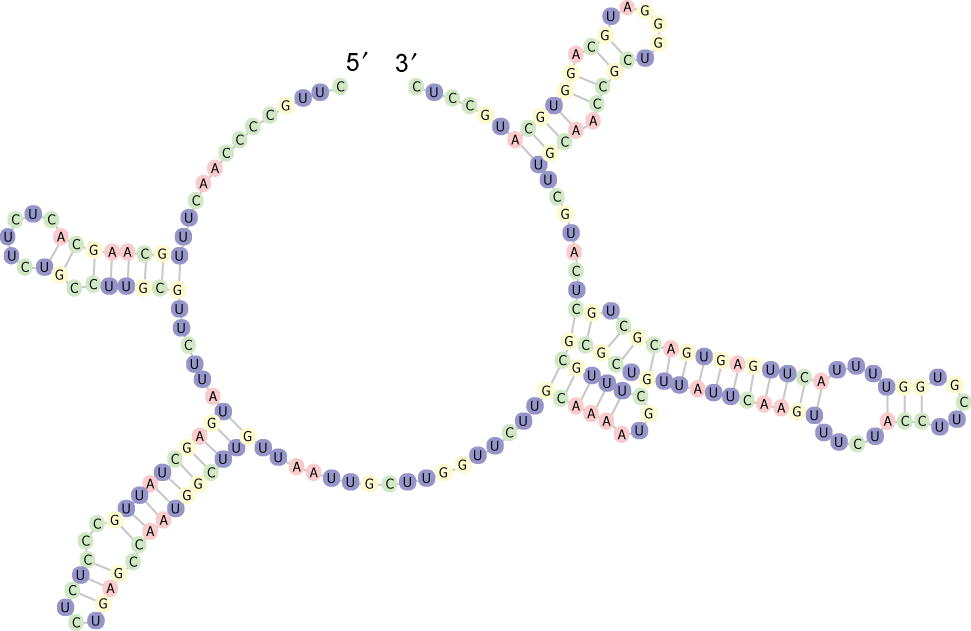


C31.5 Δ*G* -45.70 kcal/mol


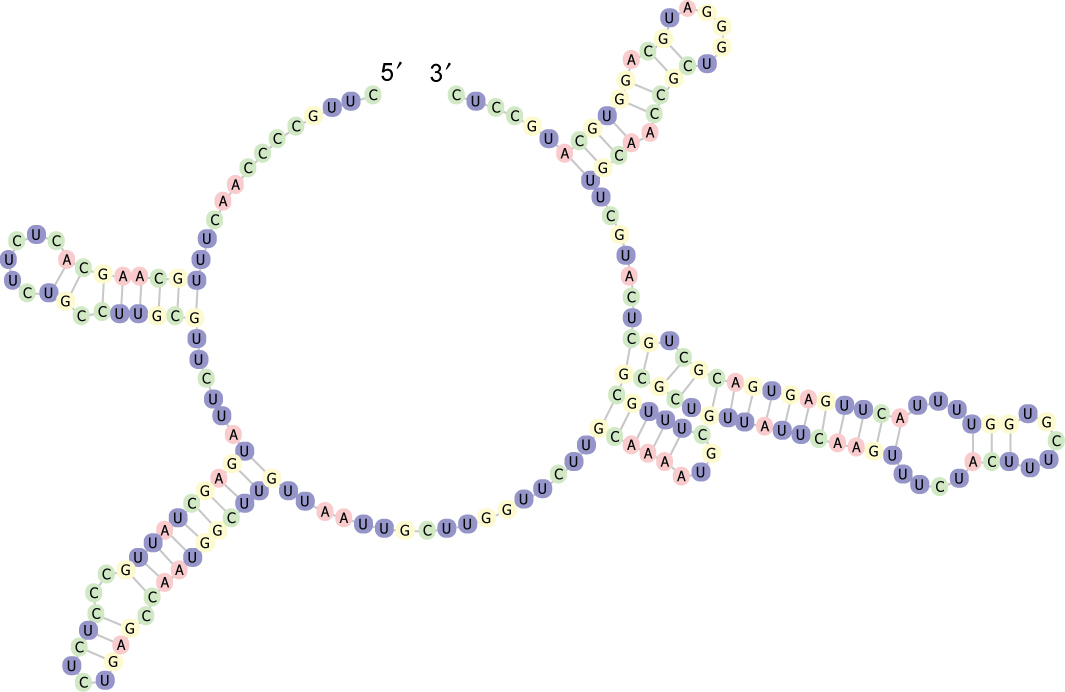


C31.6 Δ*G* -48.10 kcal/mol


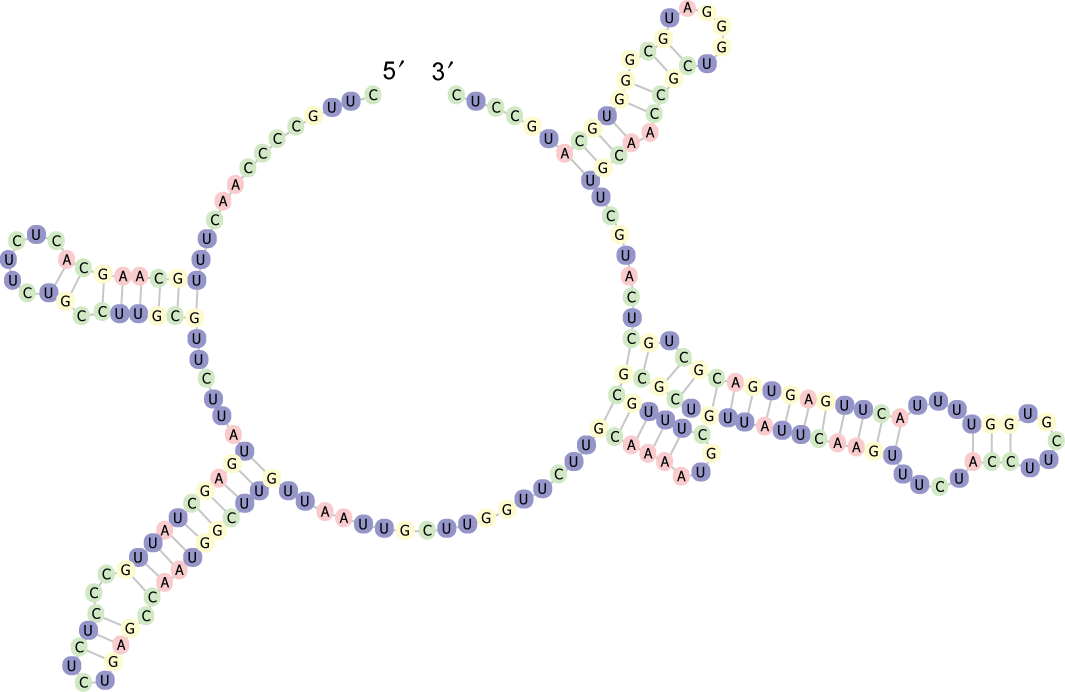


C31.9 Δ*G* -48.00 kcal/mol


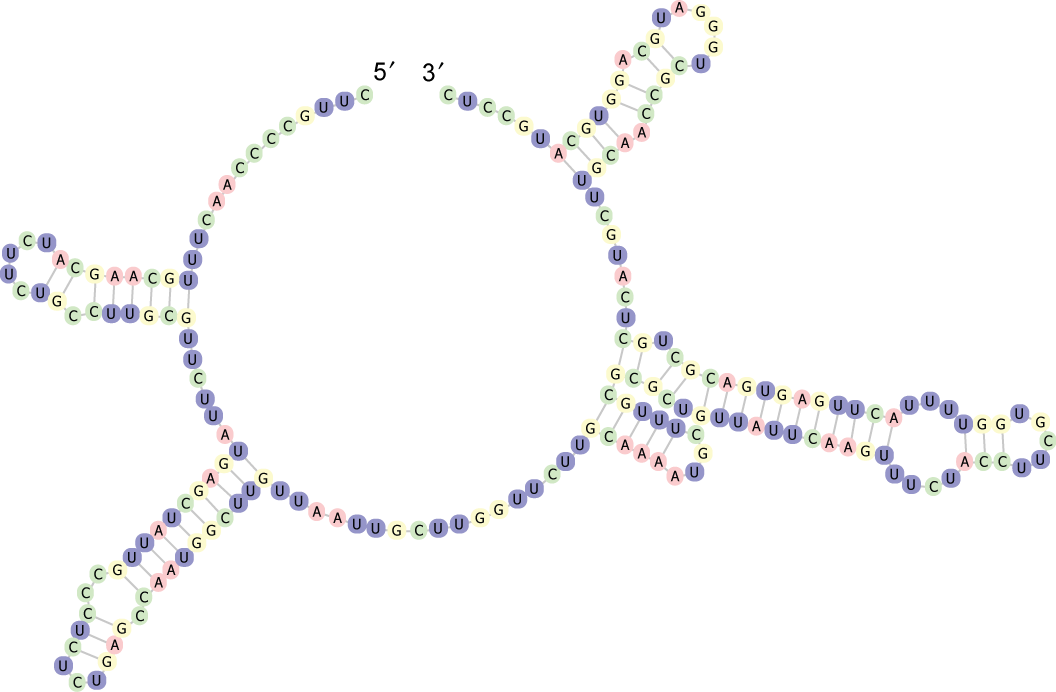


C31.10 Δ*G* -48.00 kcal/mol


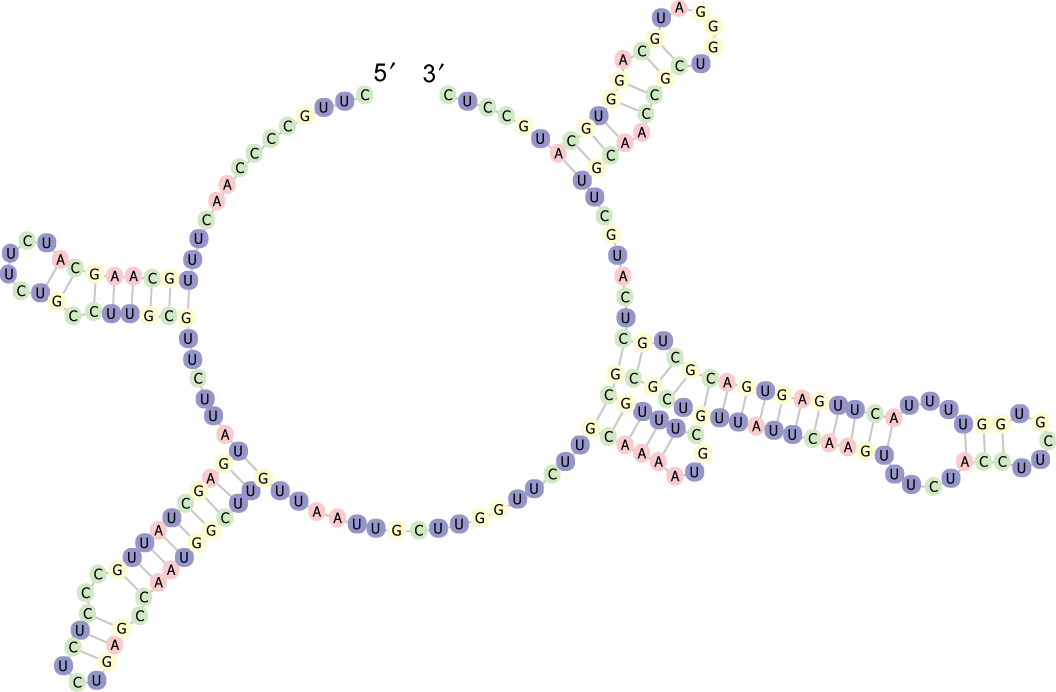


D1 Δ*G* -54.60 kcal/mol (In as D1a in Thornhill et al 2007)


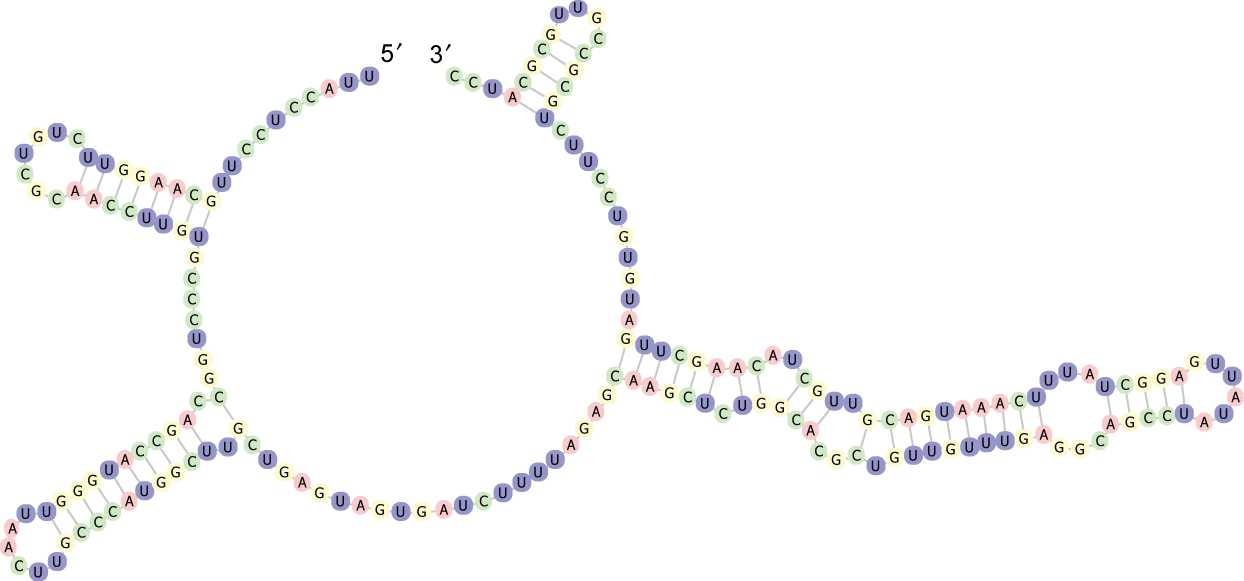


D1a Δ*G* -50.30 kcal/mol


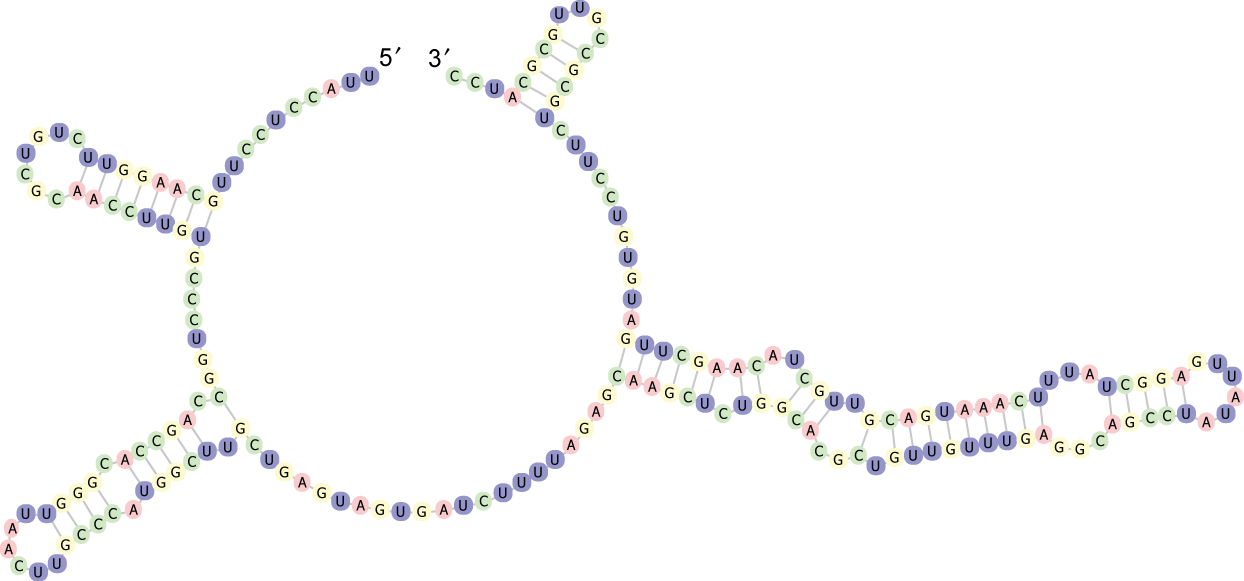


D1.6 Δ*G* -54.80 kcal/mol


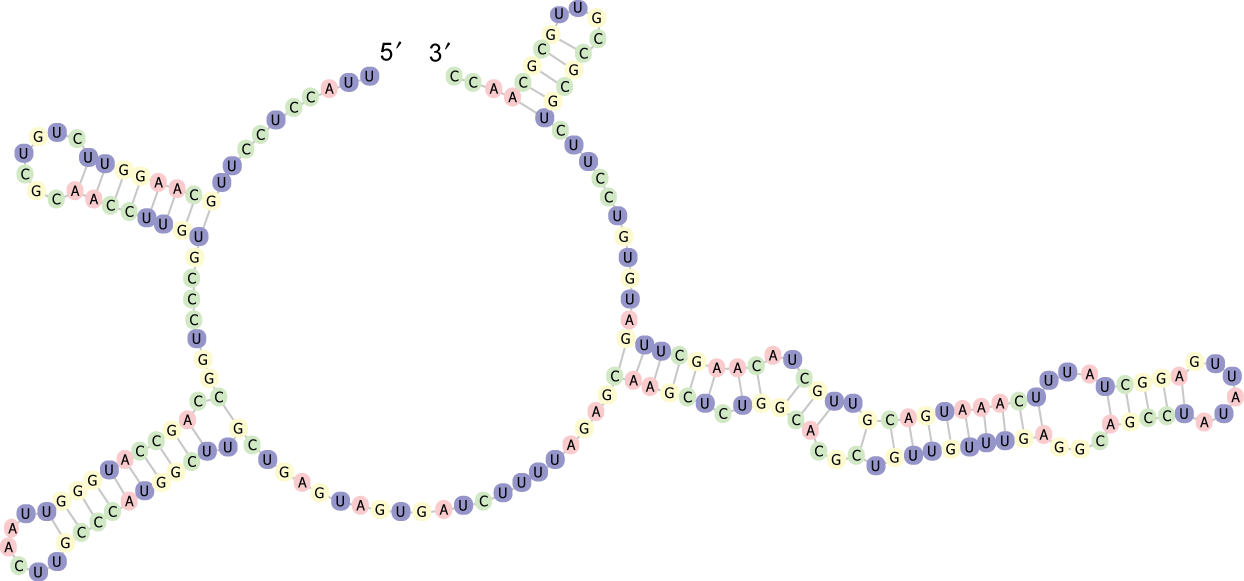

Supplement: Figure S1 — Symbiodinium ITS2 secondary structures. (DOC) [file pone.0015854.s001.doc]
